# Editorial

**DOI:** 10.1162/CPSY_e_00012

**Published:** 2017-10-01

**Authors:** Peter Dayan, Read Montague

Welcome to *Computational Psychiatry!* We are delighted to present a new open access journal devoted to “original research articles and reviews that involve the application, analysis, or invention of theoretical, computational and statistical approaches to mental function and dysfunction.” As a field, Computational Psychiatry is the modern pursuit of a new understanding of mental dysfunction, but it also has the goal of understanding broadly the sustenance of healthy mental function.

Three obvious questions come to mind: Why this field? Why now? Why start a journal?

There is now widespread recognition of the critical importance of mental health and ill health to personal development and suffering, and to society and the economy (making the comparative dearth of mental health professionals of all stripes particularly vexing). Like others, we think that novel ways of understanding psychiatric disease, accelerated by radically new sources of personal and social data, offer the possibility for revolutionary progress. However, we are also well aware that computational psychiatry is a nascent field that is still only taking its first baby steps. Nevertheless, it is by bringing together the understanding, methods, principles and enthusiasm of a thoroughly multi-disciplinary collection of experimental, theoretical and clinical scientists, that breakthroughs can be made.

Right now is the ideal time to set out on this venture. The field started more than 30 years ago, including pioneers such as the late Ralph Hoffman, and Steven Grossberg and particularly Jonathan Cohen. Over a decade ago, the first centre devoted to the topic was launched at Baylor College of Medicine. However, more recently, a number of other centres, institutes, units and courses have been created in response to the scientific interest and the broad-based demand. The many successes of the tightly-coupled experimental and theoretical field of neural reinforcement learning are providing a normative foundation for the study of dysfunctional decision-making. The resulting multi-level understanding matches well the move away from symptom-based characterizations for research and towards more fundamental information processing, cognitive and neural primitives. Equally, our new-found constant deliberate and incidental collection and leakage of personal data via smart-phones and the like offers the computationally-minded radically new paradigms to for assessing, predicting and decomposing disease.

It might not seem obvious to start a journal, even an open-access one, given the woefully increasing noise to signal ratio in academic publishing. However, we hope that rigorous reviewing, and the high standards of our publication partner, MIT Press, will prevent us from approaching too close to any unfortunate frontier. We hope the journal will also help nucleate a wider community.

We have many people to thank for helping us get to this point. These include Deanna Barch, Peter Fonagy, Helen Mayberg and Steve Hyman for forming our Advisory Board; a fantastic group of editors who span the whole field and more, and the reviewers whom they recruit and who are the backbone of any journal. A special thanks to Michael Frank and Tiago Maia for their support of the effort and the first international meeting on Computational Psychiatry in Miami in 2013. Lastly, a hearty thanks to the brave authors of the superb first submissions to the journal; Nick Lindsay and his colleagues at MIT Press for their engagement and involvement; and Cassie Carrin and before her, Justin King, for their firmly gentle steering as managing editors.

We look forward to many future papers; but most especially to making a difference.

